# Emergence and local dissemination of NDM-14-producing *Klebsiella pneumoniae* ST147: a phylodynamic and epidemiological investigation

**DOI:** 10.1128/aac.00545-26

**Published:** 2026-08-11

**Authors:** Gabriele Arcari, Mattia Carbotti, Riccardo Pavesi, Paolo Gigante, Riccardo Capuano, Domenico Caleca, Angelo Paolo Genoni, Stefano Gaiarsa, Gherard Batisti Biffignandi, Angela Kuka, Federica Novazzi, Fausto Baldanti, Nicasio Mancini

**Affiliations:** 1 Laboratory of Medical Microbiology and Virology, Department of Medicine and Technological Innovation, University of Insubria 19045, Varese, Italy; 2 Laboratory of Medical Microbiology and Virology, Ospedale di Circolo e Fondazione Macchi 18709, Varese, Italy; 3 Microbiology and Virology Unit, IRCCS Fondazione Policlinico San Matteo 18631 , Pavia, Italy; 4 Department of Biology and Biotechnology "L. Spallanzani", University of Pavia 19001, Pavia, Italy; 5 Department of Clinical, Surgical, Diagnostic and Paediatric Sciences, University of Pavia 19001, Pavia, Italy; Universita degli Studi di Roma La Sapienza, Rome, Italy

**Keywords:** *Klebsiella pneumoniae*, carbapenem resistance, NDM-14, ST147, phylodynamic analysis, outbreak investigation

## Abstract

NDM-14-producing *Klebsiella pneumoniae* ST147 has recently emerged as a high-risk clone in Europe, but its local dissemination dynamics remain poorly defined. We investigated a 6-month outbreak across three hospitals within a regional healthcare network in Lombardy, Northern Italy. Among 32 NDM-producing *K. pneumoniae*, isolated between December 2024 and May 2025, 19 belonged to ST147, including one NDM-1 producer and 18 carrying NDM-14 or its A233V variant NDM-61. Phylodynamic analysis of the ST147 NDM-14/NDM-61 genomes showed they were closely related (8–91 core SNPs) and descended from a most recent common ancestor dated to June 2024 (95% highest posterior density interval, 6 months). Reconstruction of the transmission network hints at the presence of at least three transmission clusters within the healthcare system: a major chain involving 11 of 18 cases spanning wards and facilities, a separate pair of linked cases, and 5 epidemiologically unlinked cases, suggesting missed intermediates or secondary introductions. The spread was characterized by rapid local expansion of the clone across intensive care, medical, and rehabilitation. These findings underscore the propensity of NDM-14-producing ST147 to disseminate efficiently within interconnected hospitals and highlight the need for enhanced genomic surveillance, coordinated infection control strategies, and judicious use of last-resort agents in regional healthcare networks. In a global phylogenetic context, the Italian isolates clustered within a broader NDM-14 ST147 population composed of several geographically distinct clades associated with France, Italy, Spain, Morocco, and the United Kingdom.

## INTRODUCTION

Carbapenem-resistant *Klebsiella pneumoniae* (CRKP) is one of the most problematic causes of healthcare-associated infections in Europe, with Italy among the countries reporting the highest incidence and attributable mortality (1). Recent analyses report an increasing epidemiological role of metallo-beta-lactamase (MBL)-producing *K. pneumoniae*, with the New Delhi MBL (NDM) accounting for a substantial proportion of bloodstream and invasive infections in Italian hospitals, where regional outbreaks and endemic transmission have been repeatedly documented (2, 3). Against this background, the emergence of successful “high-risk” lineages capable of spreading across institutions and borders is of particular concern. Sequence type 147 (ST147) has been recognized as one of these high-risk clones, combining beta-lactam resistance with a propensity for inter-facility dissemination and long-term persistence in colonized patients (4). Alongside the most common NDM variants (NDM-1 and NDM-5), new variants have emerged (5). NDM-14 is an NDM-1 variant characterized by a D130G substitution (6). These NDM-14-producing ST147 isolates have recently been described as a cause of outbreak in France (7), Morocco (8), and Spain (9), but no case has been reported so far from Italy.

In this study, we describe the rapid spread of this ST147 NDM-14 clone within the *ASST Sette Laghi* (ASST7L) healthcare network in Lombardy, Northern Italy. The ASST7L is one of the two Local Health Authorities in the Varese province, and it is organized into seven distinct hospitals with an overall bed capacity of 1,300. Here, by integrating whole-genome sequencing (WGS), phylodynamic, and transmission network analysis including patient-movement data, we aimed to reconstruct the evolutionary history and local spread of this clone in the Varese area, placing it within the global NDM-producing ST147 context.

## RESULTS

From 1 December 2024 to 31 May 2025, a total of 36 NDM-producing *K. pneumoniae* were isolated from the same number of patients, either hospitalized in three out of the seven ASST7L hospitals (35/36 patients, hospitalized in 15 different wards) or presenting as outpatients (1/36) (Table S1). Upon thawing, it was not possible to resuscitate 4 out of 36 NDM-producing *K. pneumoniae*, while the remaining 32 were subjected to WGS. Among these, three did not harbor the *bla*_NDM_ gene (possibly due to plasmid loss or the co-presence of other NDM-encoding microorganisms in the same sample), and the majority of isolates (19/29) belonged to ST147 (Table S1).

### Antimicrobial resistance patterns and main features of NDM-14-producing *Klebsiella pneumoniae* ST147

In the analyzed period, two discernible NDM-producing ST147 *K. pneumoniae* lineages could be identified as circulating in the ASST7L. The first was represented by a single isolate encoding NDM-1 and characterized by an OmpK35 truncation at 46% of wild-type length, wild-type OmpK36, and a KL20 capsule type. The second, instead, included a total of 18 isolates, all encoding NDM-14 or, in one case, its A233V variant NDM-61. These isolates were characterized by an OmpK35 truncated at 42% of wild type by a premature stop codon caused by a seven-nucleotide frameshift mutation, wild-type OmpK36, and a KL64 capsule type and shared a highly conserved acquired resistome and chromosomal background. The NDM-1-producing ST147 isolate was highly divergent from the NDM-14/61 clade, differing by 2,218–2,259 core-genome SNPs and was hence not considered as part of the same outbreak.

The presence of a wide array of resistance genes was reflected in the antimicrobial resistance profile, with all isolates demonstrating uniform resistance to carbapenems and new beta-lactam/beta-lactamase inhibitor combinations while remaining susceptible to aztreonam-avibactam; in contrast, cefiderocol minimum inhibitory concentrations (MICs) varied widely from 0.125 to 4 mg/L (Table S2). Potential determinants of cefiderocol MIC variability were investigated via the CefiderocolFinder tool (10), which did not identify any difference among the genomes. All isolates were resistant to aminoglycosides, fluoroquinolones, and folate pathway inhibitors as well, but susceptible to colistin and displaying low MICs for third-generation tetracyclines (tigecycline and eravacycline).

All NDM-14/NDM-61-producing ST147 isolates shared an identical plasmid replicon profile, including the ColRNAI, IncFIB(Mar), and IncHI1B from in the pNDM-Mar variant, IncFIB(pKPHS1), IncFIB(pQil), and IncR replicons. While the highly conserved replicon content suggests circulation of a conserved *bla*_NDM_-bearing plasmid backbone, no long-read sequencing approach was carried out, making it impossible to unequivocally identify the plasmid encoding NDM.

The D130G substitution sets apart NDM-14 from NDM-1 and, besides enhancing catalytic activity toward carbapenems, it is not consistently detected by lateral flow immunoassays (LFIAs) commonly used for rapid carbapenemase screening and was identified only by real-time PCR tests.

In addition to the resistome, the virulence profile was homogeneous as well, with all genomes carrying the yersiniabactin locus *ybt9* assigned to the ybST173 with two locus variants on an integrative conjugative element ICE*Kp*3-like and the aerobactin siderophore biosynthesis gene cluster *iuc1*.

While the regulator of mucoid phenotype *rmpA* was not detected, all isolates systematically displayed a disruption in the *rmpA2* gene characterized by premature stop codons occurring in 3 isolates at 55% of wild-type length and in 16 isolates at 60% of wild-type length, which are predicted to abolish its function of capsule overexpression leading to a hypermucoviscous phenotype (2, 11). No other major virulence determinant, such as salmochelin or colibactin, was detected.

### Local spread and diffusion of NDM-14-producing *Klebsiella pneumoniae* ST147 in three hospitals

Among the 32 sequenced NDM-producing *K. pneumoniae* isolates, 18 produced NDM-14 or its A233V variant NDM-61, and all belonged to ST147. During the same period, only another NDM-producing ST147 isolate was identified.

The majority of the latter isolates were sampled from patients hospitalized in wards of the Varese tertiary-care university hospital (15/18), fewer cases were detected in the secondary-care hospitals of Tradate (2/18) and Angera (1/18), and one case was detected from an outpatient (1/18) with no previous hospitalization within the ASST7L healthcare system nor antimicrobial treatment. Cases clustered in high-acuity settings, particularly intensive care units, medical wards, neurosurgical units, and rehabilitation facilities (Table S1). All isolates displayed an identical LIN code profile (12) up to the seventh bin (0_0_197_0_4_1_0) except for isolate VA_10, the only one sampled from the Angera hospital, which shared the profile up to the sixth bin.

**Fig 1:**
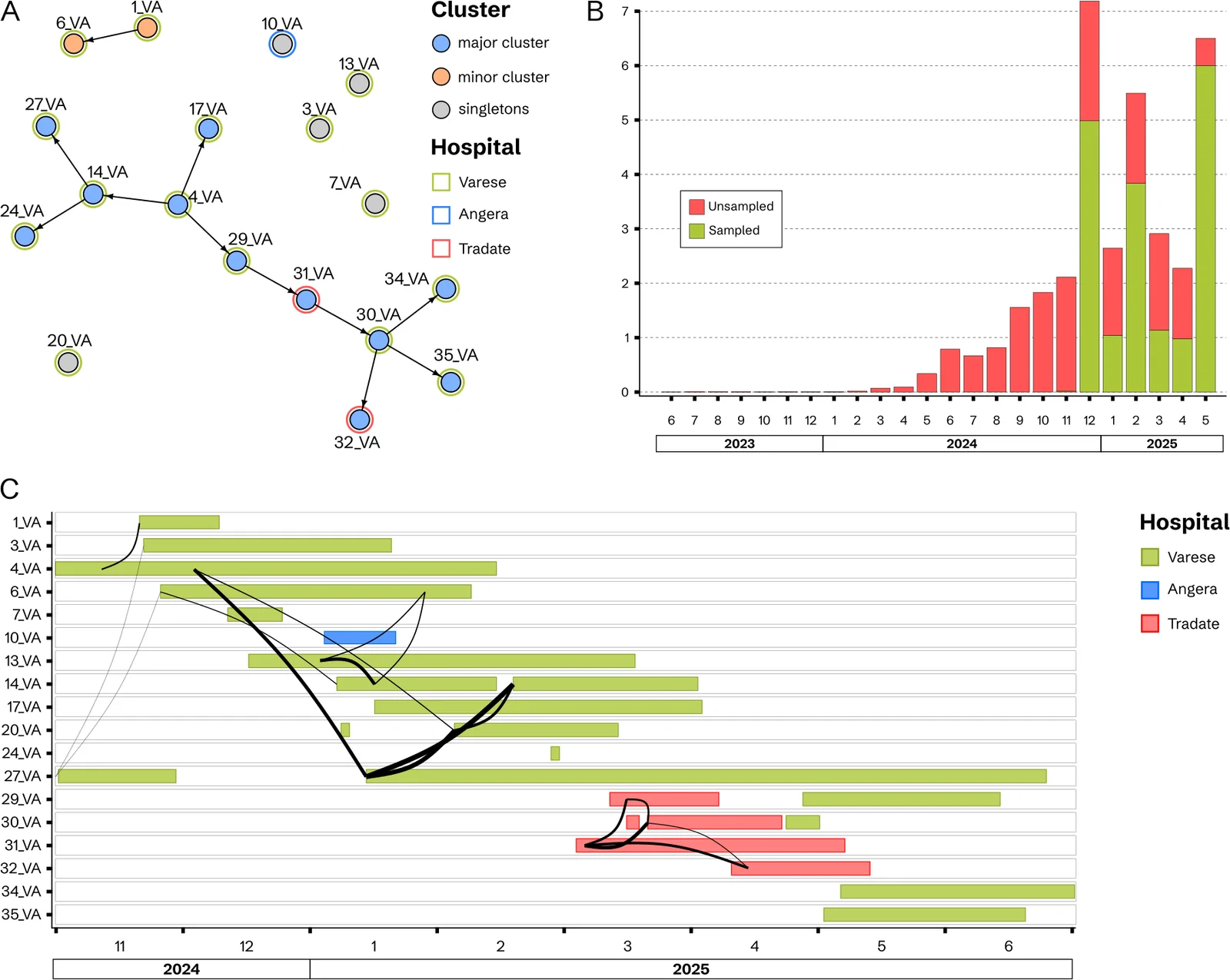
Transmission network and temporal distribution of the analyzed ST147 *Klebsiella pneumoniae* cases. (**A**) Network graph showing inferred patient-to-patient transmission links for the 18 ST147 *Klebsiella pneumoniae* cases. Nodes represent individual patients (labeled by patient ID) and are colored according to cluster assignment: major transmission cluster (blue), minor transmission cluster (orange), or unconnected introductions (gray). Edges connecting nodes represent probable transmission links inferred by Outbreaker2 (13) and TransPhylo (14) analyses. (**B**) Stacked bar chart showing the temporal distribution of NDM-14-producing ST147 *K. pneumoniae* cases binned by month. Green bars represent sampled and sequenced isolates, while red bars represent isolates inferred to belong to the same transmission cluster by posterior analysis of TransPhylo (14) but not sampled or sequenced. (**C**) Gantt chart depicting temporal hospital occupancy for the 18 patients with NDM-14-producing ST147. Each horizontal bar represents the stay in a specific hospital setting and is colored according to city. Black connecting lines overlay the inferred transmission tree, with line thickness proportional to the posterior probability of transmission between patients as estimated by TransPhylo (14).

Transmission modeling allowed to infer how most cases of this cluster (11/18) were linked in a major diffusion within one or two transmission generations, while in a minority of cases no significant hypothesis of transmission could be drawn (5/18), or a lone cluster was observed (2/18) (Fig. 1A).

Dated phylogeny-based inference of the transmission tree highlighted the potential presence of up to three unsampled isolates starting from September 2024, hence before the analyzed period, and up to nine unsampled isolates in the timeframe ranging from December 2024 to the end of May 2025. While it is possible to hypothesize that a portion of the four NDM-producing *K. pneumoniae* isolates which were not resuscitated could have been assigned to this cluster, transmission network estimates suggest the existence of undetected intermediates in the outbreak chain (Fig. 1B).

Analysis of patient-movement data showed that several individuals with ST147 NDM-14 had overlapping stays in the same ward or in closely connected units within a 5-day window, supporting the occurrence of in-hospital transmission (Fig. 1C). Notably, some patients moved between hospitals, such as those transferred from Tradate to Varese for higher-level care or from Varese to rehabilitation wards, creating potential bridges for inter-facility dissemination.

Core genome SNP distances within this clade ranged from 8 to 91 SNPs (mean 31, standard deviation 17; Fig. S1), reflecting the expected variation accumulated over approximately 6 months of nosocomial circulation. The substitution rate was estimated to be at 6.13 × 10^−6^ substitutions/site/year (95% highest posterior density, HPD, interval 4.41–11.57). This was higher when compared with previously reported rates for ST147 *K. pneumoniae* (7, 15) but consistent with nosocomial outbreaks. Indeed, the short timeframe of sampling interferes with the intensity and reliability of the molecular clock model, which in this case did not show a significant temporal signal (*R*^2^ = 0.12, *P* = 0.09).

Phylodynamic analysis estimated the MRCA of these NDM-14 ST147 strains in June 2024 (95% HPD, interval January 2024–September 2024), approximately 6 months prior to the first isolate sampled from our healthcare network, suggesting either prior cryptic circulation within the province or external introduction of an already-established lineage. However, to the best of our knowledge, no other case was reported from other Lombardy region provinces

The presence of the yersiniabactin *ybt*9 locus, OmpK35 mutations, and mobilome content are consistent with ST147 isolates circulating in Italy (2, 11).

### Global context and circulation of the NDM-14-producing *Klebsiella pneumoniae* ST147 lineage

ST147 isolates form multiple geographic and temporal clusters and are distributed across Europe, Asia, Africa, and the Americas (15). This sequence type has been repeatedly associated with healthcare-associated outbreaks, particularly in post-acute care settings, primarily owing to carbapenemase production and, in some lineages, hypervirulence elements (3).

**Fig 2:**
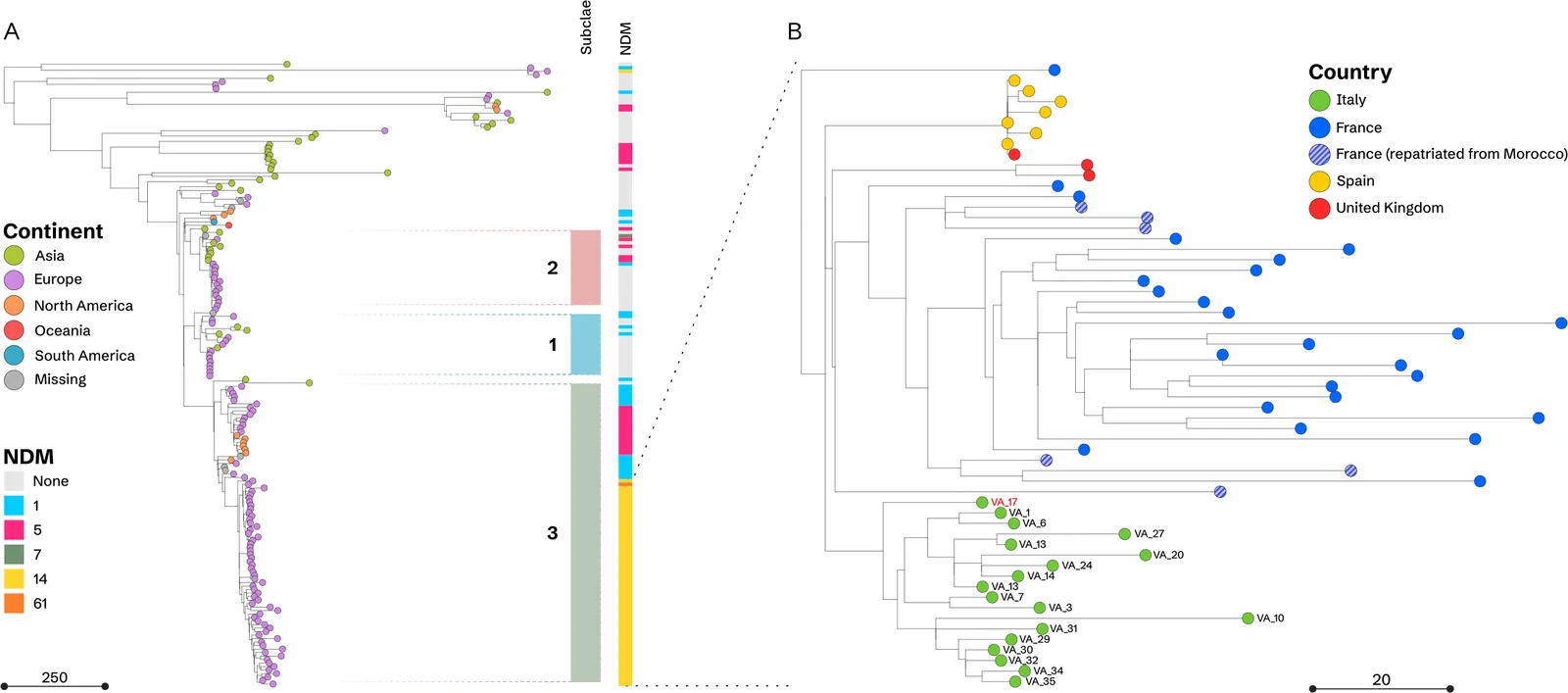
Global distribution of ST147 *Klebsiella pneumoniae*. (**A**) Maximum-likelihood phylogenetic tree of 178 ST147 *Klebsiella pneumoniae* genomes, including the 18 ST147, 121 complete ST147 genomes (3 of which encoding NDM-14), and 39 NDM-14 genomes sequenced at a contig level from the RefSeq database. Tips are colored by continent of isolation (Asia: green; Europe: purple; North America: orange; Oceania: red; South America: teal; and missing data: gray). Metadata on the right-side bar indicate the belonging to one of the ST147-KL64 subclades 1 and 2 (as per reference 15) and subclade 3 described here and the NDM variant carried by each isolate (none: white, NDM-1: cyan, NDM-5: magenta, NDM-7: dark green, NDM-14: yellow, and NDM-61: orange). (**B**) Midpoint rooted phylogeny of the NDM-14-like producing *K. pneumoniae* identified globally as of December 2025 and from this study. Tips are colored by country of isolation (Italy: green; France: blue; French patients repatriated from Morocco (7): blue with pink stripes; Spain: yellow; and United Kingdom: red). The NDM-61-producing isolate is highlighted by a red label.

When positioned within the global ST147 population (complete NCBI RefSeq genomes as of 1 December 2025), the 50 NDM-14-producing genomes cluster in a monophyletic group with reduced genetic diversity (Fig. 2A; Data set S1 and S2). All NDM-14 isolates were characterized by the KL64 capsule type, the yersiniabactin *ybt*9 locus, and an OmpK35 truncation caused by a seven-nucleotide frameshift.

The ST147-KL64 subclades 1 and 2 are characterized by *ybt*16/ICE*Kp*12 coupled with an OmpK35 truncated by a two-nucleotide frameshift mutation and by *ybt*10/ICE*Kp*4 coupled with an OmpK35 disrupted by IS*Ecp*1. The elements observed in NDM-14-producing isolates support the definition of a novel ST147-KL64 subclade, which was named “subclade 3” (Table 1).

**TABLE 1:** Main genetic features defining the three ST147-KL64 subclades

ST147-KL64 subclade	*ybt* lineage and ICE*Kp* structure	OmpK35 alteration
Subclade 1	*ybt*16 associated with ICE*Kp*12	Two-nucleotide frameshift mutation
Subclade 2	*ybt*10 associated with ICE*Kp*4	Gene truncation by ISE*cp1* insertion
Subclade 3	*ybt*9 associated with ICE*Kp*3	Seven-nucleotide frameshift mutation

The NDM-14 variant has been identified in 42 *K. pneumoniae* genomes globally as of 1 December 2025, with the vast majority (41/42) belonging to ST147. As concerns NDM-61, instead, this variant has been identified only one other time in an ST11 *K. pneumoniae* from Greece (16). The geographic distribution of NDM-14-like producing ST147 shows a geographically structured clade distribution, with distinct clusters associated with France (particularly from Morocco-linked importation events in 2021–2022), Spain, the United Kingdom, and Italy (Fig. 2B).

Indeed, the ASST7L cluster is distinct from what was previously described and finds the closest genome in one isolate sampled in the Grand Est region not belonging to any major cluster in the French epidemiology (isolate 299A10, RefSeq assembly GCF_028655615.1).

This pattern is consistent with the presence of a common NDM-14-producing ST147 ancestor, plausibly originating from the Casablanca region in Morocco (7, 8), which was introduced multiple times in different European regions. Each European occurrence then underwent local amplification, supported by inadequate genomic surveillance systems.

## DISCUSSION

Two main subclades have been described in the structure of the ST147-KL64 clade: subclade 1 (characterized by an *ybt*16 carried by an ICE*Kp*12 and an OmpK35 truncated by a premature stop codon caused by a two-nucleotide frameshift mutation) and subclade 2 (characterized by an *ybt*10 carried by an ICE*Kp*4 and an altered OmpK35 gene disrupted by IS*Ecp1-bla*_CTX-M-15_).

Given the presence of a KL64 capsular antigen, a different *ybt* locus, and OmpK35 truncation, these isolates can hence be defined as part of the ST147-KL64 subclade 3, and this study describes the emergence and rapid expansion of this subclade (15), within the ASST7L healthcare network in Lombardy, Italy. Over 6 months, 18 patients were identified with NDM-14-producing ST147, and phylodynamic analysis dated the most recent common ancestor (MRCA) to June 2024 (95% HPD, January 2024–September 2024). Since this occurred approximately 6 months before the first isolate was detected in the ASST7L healthcare network, it is possible to hypothesize a priori cryptic circulation in the area before recognition. While the modest number of sequenced isolates inevitably reduced the precision of phylodynamic and transmission inferences, all NDM-producing isolates detected in the ASST7L network during the study period were sequenced and combined with ward-level patient-movement data. Specifically, the integration of epidemiological and transmission modeling revealed that 11 of 18 cases were linked to a single chain by patient-to-patient transmission within and between hospitals, 2 cases were linked among themselves, and 5 cases remained unexplained, suggesting either undetected transmission or repeated introductions. Although NDM isolates generally display high MICs for carbapenems, it is not possible to exclude that carbapenemase producers with low MICs might have bypassed our diagnostic flowchart targeting only isolates with non-susceptible at standard dosage carbapenem phenotype (i.e., meropenem and/or imipenem >2 mg/L).

Our findings align with the French NDM-14-producing ST147 outbreak documented in 2021–2023 (7) and the Canary Islands one in 2023–2025 (9). However, the Varese area cluster forms a distinct monophyletic lineage with recent divergence from the broader European NDM-14 ST147 population, indicating possible independent or parallel introduction events rather than a direct dissemination from France. This pattern shows separate clusters in Spain, the United Kingdom, and Italy, and is consistent with multiple introductions of NDM-14 into distinct ST147 backgrounds across Europe, likely originating from the Casablanca region of Morocco as previously documented (7).

The observed spread of this subclade may reflect a combination of multidrug resistance, transmissibility in hospital settings, and a virulence background that includes yersiniabactin and aerobactin loci.

The three hospitals in Varese, Tradate, and Angera share patients and staff, creating natural routes for dissemination. Priority interventions include systematic screening for carbapenemase-producing *K. pneumoniae* in high-risk wards, contact precautions, and inter-facility communication at patient transfers. Given the preserved activity of aztreonam-avibactam and, with a high MIC variability, cefiderocol, antimicrobial stewardship should prioritize their appropriate use for confirmed NDM-producing infections. Furthermore, the association of resistant phenotype, coupled with the presented issues in NDM-14 detection using rapid diagnostic chromatography-based methods, calls attention to the importance of genomic surveillance frameworks and their integration with infection-control measures to detect and limit the amplification and dissemination of multidrug-resistant pathogens.

## MATERIALS AND METHODS

### Study setting and data collection

The Laboratory of Medical Microbiology and Virology of the Varese University Hospital serves a population of approximately 500,000 people within the ASST7L healthcare network, which includes seven different hospitals. Reports of NDM-producing *K. pneumoniae* from both hospitalized and community patients in the period December 2024–June 2025 were included, and the respective isolates were stored at −80°C for further analyses. Pseudonymized patient data associated with each isolate were collected for the analysis and included age, gender, specimen type, date of isolation, and geographical location of the originating microbiology laboratory.

### Bacterial identification and antimicrobial susceptibility testing

Bacterial isolates were identified by MALDI-ToF MS (VITEK-MS, bioMérieux, Marcy-l'Étoile, France), antimicrobial susceptibility was tested by Vitek2 system (bioMérieux, using the 438 and XN26 cards), and interpreting minimum inhibitory concentration (MIC) values in accordance with EUCAST breakpoint tables (https://www.eucast.org/bacteria/clinical-breakpoints-and-interpretation/clinical-breakpoint-tables/). Up until March 2025, all *K. pneumoniae* isolates displaying a carbapenem-resistant phenotype were tested for the presence of genes encoding the most common carbapenemases (i.e., VIM, IMP, KPC, OXA-48, and NDM) using the real-time PCR assay Xpert Carba-R kit (Cepheid). Starting from March 2025, the KPC/IMP/NDM/VIM/OXA-48 Combo Test Kit (Medomics-dx, Nanjing, China) Lateral Flow Immunoassay (LFIA) was introduced as a primary test to identify the production of the respective carbapenemases. In case of negative LFIA in carbapenem-resistant isolates, the real-time PCR assay Xpert Carba-R was carried out as a second-line test to rule out potential false negatives. The Ezer Carbapenemase Rapid Test (Servizi Diagnostici S.r.L., Ciampino, Italy) was introduced as a second LFIA in October 2025; hence, its performances in the detection of NDM-14 were purely retrospective on isolates known to be positive.

Aztreonam-avibactam MIC was tested by gradient strip test method (Liofilchem, Roseto degli Abruzzi, Italy), while cefiderocol and colistin MICs were calculated by broth microdilution, using compact antimicrobial susceptibility panel (ComASP; Liofilchem) and Sensititre Gram Negative Susceptibility Testing Plate EUMDRXXF (Thermo Fisher Scientific, Waltham, MA, USA).

### Whole-genome sequencing and typing

**Fig 3:**
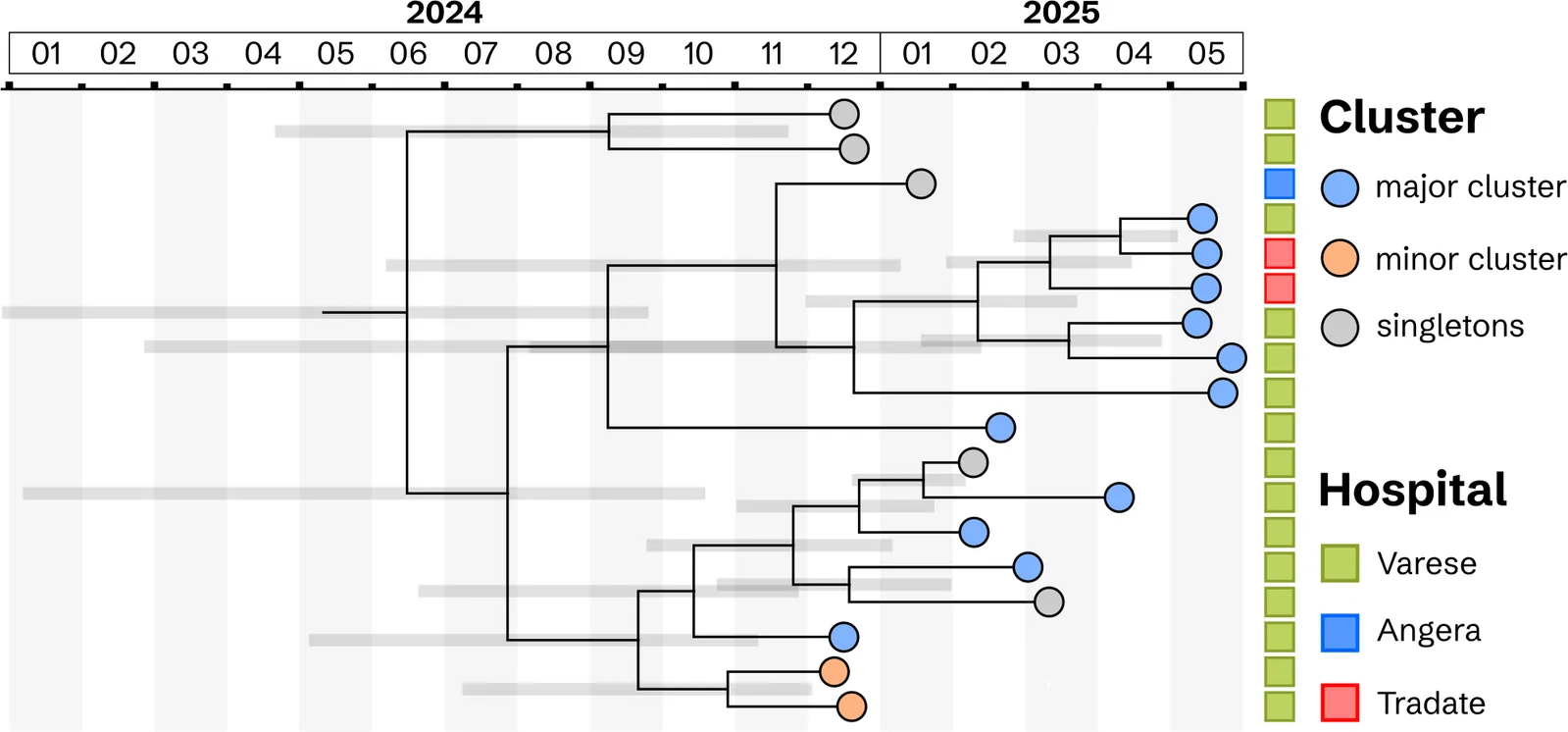
Time-scaled phylogeny of *Klebsiella pneumoniae* ST147. Time-calibrated phylogenetic analysis of the 17 NDM-14 and the single NDM-61-producing ST147 isolates recovered from the ASST Sette Laghi network. Branch lengths are proportional to time (in years), and light gray bars at each node indicate the 95% highest posterior density (HPD) interval for the branching date. Tips are colored according to cluster assignment: major transmission cluster (blue), minor transmission cluster (orange), or unconnected introductions (gray). Metadata are colored according to sampling city. The tree is rooted at the estimated most recent common ancestor dated to June 2024 (95% HPD, interval January 2024–September 2024), and the mean substitution rate was estimated to be 6.13 × 10^−6^ substitutions per site per year.

After overnight growth, DNA from NDM-producing *K. pneumoniae* was purified from bacterial strains using the ELITe InGenius SP 200, paired-end libraries were generated using the Nextera XT DNA sample preparation kit (Illumina Inc, San Diego, CA, USA), and sequenced in-house using the Illumina MiSeq instrument with 2 × 250 PE protocol (Illumina Inc). *De novo* assembly of Illumina reads was performed using shovill v1.1.0 (https://github.com/tseemann/shovill). Species confirmation, multilocus sequence typing, and detection of antimicrobial resistance and virulence genes were performed using Kleborate (v3.0) (17), while capsular and O antigen serotype prediction was performed using Kaptive (18). LIN code definition was carried out using Pathogenwatch (https://pathogen.watch/) (12). Plasmid incompatibility groups were identified with PlasmidFinder (19). Phylogenetic relationships were assessed among all ST147 NDM-producing *K. pneumoniae* recovered from our institution (19 genomes) and among those carrying *bla*_NDM-14_ and single-nucleotide variants (18 genomes, Fig. 3; Fig. S1). In both cases, whole-genome alignments were generated using SKA2 (Split Kmer Analysis [20]), recombinant regions were detected and removed with Gubbins (21), and maximum-likelihood phylogenies were inferred with IQ-TREE v2.1.4 (22).

### Molecular clock and transmission network reconstruction analyses

Evolutionary rates and the age of the most recent common ancestor (MRCA) were estimated using the BactDating R package (23). Both additive relaxed clock (arc) and continuous additive relaxed clock (carc) models were used to account for rate variation among lineages, with the carc model displaying a slightly superior fit (deviance information criterion, DIC, for carc = 261.28 and for arc = 266.35). To further assess the fit of the carc model, this was compared with a constant-rate model based on the mean date of sampling (DIC for carc = 261.28 and for constant-rate model = 301.65). Parameters jointly estimated included the molecular clock rate (*μ*), rate variation parameter (*α*), and standard deviation (*σ*), allowing for the update of all these parameters and the tree root. The Markov chain Monte Carlo (MCMC) was run for 10^7^ iterations. The MCMC showed adequate mixing, with effective sample sizes (ESS) after convergence of *μ* = 574.0875, *σ* = 590.3487, and *α* = 501.0000.

Transmission dynamics were inferred using Outbreaker2 (13) and TransPhylo (14) R packages. Both methods applied gamma-distributed serial interval parameters derived from reported *K. pneumoniae* transmission studies, corresponding to a mean of 4 days and a standard deviation of 2 days (*γ* shape = 8.16; scale = 0.49). The initial parameter settings were *π* = 0.7 (sampling proportion), *ν* = 0.2 (proportion of known locations), Reff = 1.0 (effective reproduction number), and *P* = 0.67 (reporting probability). Each MCMC run comprised 100,000 iterations with a thinning interval of 50. Using a 50% burn-in, the MCMC showed adequate mixing with ESS after convergence of *π* = 141.1, negative branching parameter (neg) = 107.2, mean reproduction number (off.r) = 96.9, and offspring distribution parameter (off.p) = 129.3.

Epidemiological linkage was defined as temporal and spatial overlap in ward occupancy within a 5-day window around each isolation date, as derived from hospital admission and discharge data. Patient movements between wards and facilities were obtained as pseudonymized records and plotted to visualize possible transmission events using HAIviz (24).

### Global analysis of NDM-14-producing *Klebsiella pneumoniae* (and of ST147)

To explore the worldwide occurrence of NDM-14-producing *K. pneumoniae*, on 1 December 2025, a BLASTN search was performed using the *bla*_NDM-14_ reference sequence (accession number NG_049331.1) against all genomes in the RefSeq database regardless of the assembly level. Search parameters were adjusted, with *max target sequences* set to 500 and *word size* to 54. Forty-two *K. pneumoniae* genomes with 100% sequence identity across the full *bla*_NDM-14_ gene were identified, and all associated assemblies were retrieved for comparative genomic analysis. Metadata including country, isolation source, and collection date were extracted when available from the RefSeq records and supplemented with information from published manuscripts (7, 9).

For global ST147 genomic context, all complete *K. pneumoniae* sequences were downloaded from NCBI RefSeq Genome Database, which as of 1 December 2025 included 1,847 genomes. After ST assignment, phylogenetic relationships among all complete ST147 genomes, all NDM-14-encoding genomes, and the 18 ST147 NDM-14-producing isolates from this study were inferred using SKA2 (20), Gubbins (21), and IQ-TREE (22) with the same parameters described above.

## Data Availability

Sequences of NDM-producing *Klebsiella pneumoniae* analyzed in this study have been deposited in the NCBI GenBank Database under BioProject PRJNA1419765.

## SUPPLEMENTAL MATERIAL

Fig. S1 aac.00545-26-s0001.tiff Single nucleotide polymorphisms clustermap matrix.

Data set S1 aac.00545-26-s0002.xlsx Replicon content of the 173 ST147 *Klebsiella pneumoniae* genomes analyzed in this study.

Data set S2 aac.00545-26-s0003.xlsx Resistance gene content of the 173 ST147 *Klebsiella pneumoniae* genomes analyzed in this study.

Supplemental material aac.00545-26-s0004.docx Legend for Fig. S1.

Table S1 aac.00545-26-s0005.docx Main characteristics of the NDM-producing *Klebsiella pneumoniae* isolates from the analyzed period.

Table S2 aac.00545-26-s0006.docx Antimicrobial susceptibility profiles and resistance genes content of the NDM-producing ST147 *Klebsiella pneumoniae*.

ASM does not own the copyrights to Supplemental Material that may be linked to, or accessed through, an article. The authors have granted ASM a non-exclusive, world-wide license to publish the Supplemental Material files. Please contact the corresponding author directly for reuse.
