# Supplementary material for: Emergence and local dissemination of NDM-14-producing Klebsiella pneumoniae ST147: a phylodynamic and epidemiological investigation
Source: Antimicrob Agents Chemother. 2026 Aug 11;70(9):e00545-26. doi: 10.1128/aac.00545-26 (PMC13712845; doi:10.1128/aac.00545-26)
Supplement: Supplementary file 1 [file aac.00545-26-s0004.docx]

**Supplementary Figure 1 Genomic relatedness of sequenced NDM-producing ST147 *Klebsiella pneumoniae* isolates**

Single nucleotide polymorphisms clustermap matrix focused on the 18 NDM-14/NDM-61 producing isolates.
