# Supplementary material for: Emergence and local dissemination of NDM-14-producing Klebsiella pneumoniae ST147: a phylodynamic and epidemiological investigation
Source: Antimicrob Agents Chemother. 2026 Aug 11;70(9):e00545-26. doi: 10.1128/aac.00545-26 (PMC13712845; doi:10.1128/aac.00545-26)
Supplement: Supplementary file 2 [file aac.00545-26-s0005.docx]

| **Sampling** | **Specimen** | **City** | **Ward** | **ID** | **ST** | **NDM** | **Cluster** |
| --- | --- | --- | --- | --- | --- | --- | --- |
| 2024-12-02 | Rectal Swab | Varese | Medicine - 2 | 1_VA | 147 | 14 | Minor |
| 2024-12-05 | Urine | Varese | Emergency Room | 2_VA | 512 | 1 | NA |
| 2024-12-06 | Wound | Varese | Vascular surgery | 3_VA | 147 | 14 | Singleton |
| 2024-12-06 | RT | Varese | Physical therapy | 4_VA | 147 | 14 | Major |
| 2024-12-08 | Urine | Varese | Emergency Room | 5_VA* | NA | NA | NA |
| 2024-12-09 | Rectal Swab | Varese | Cardiovascular ICU | 6_VA | 147 | 14 | Minor |
| 2024-12-10 | Wound | Varese | Thoracic surgery | 7_VA | 147 | 14 | Singleton |
| 2024-12-10 | Urine | Varese | General surgery | 8_VA | 147 | 1 | NA |
| 2024-12-20 | Urine | Varese | Nursing home | 9_VA | 6668 | 1 | NA |
| 2025-01-06 | Wound | Angera | Medicine | 10_VA | 147 | 14 | Singleton |
| 2025-01-09 | RT | Varese | Physical therapy | 11_VA | 6668 | 1 | NA |
| 2025-01-15 | Urine | Tradate | Physical therapy | 12_VA | 6668 | 1 | NA |
| 2025-01-28 | Rectal Swab | Varese | Medicine - 2 | 13_VA | 147 | 14 | Singleton |
| 2025-01-28 | RT | Varese | Medicine - 2 | 14_VA | 147 | 14 | Major |
| 2025-02-06 | Rectal Swab | Varese | Medicine - 2 | 15_VA | 512 | - | NA |
| 2025-02-06 | Rectal Swab | Varese | Physical therapy | 16_VA | 11 | 1 | NA |
| 2025-02-11 | Rectal Swab | Varese | ObGyn | 17_VA | 147 | 61 | Major |
| 2025-02-14 | Urine | Varese | Emergency Room | 18_VA | 6668 | 1 | NA |
| 2025-02-14 | Urine | Varese | Medicine - 1 | 19_VA | 512 | 1 | NA |
| 2025-02-18 | Rectal Swab | Varese | Physical therapy | 20_VA | 147 | 14 | Singleton |
| 2025-02-20 | Urine | Varese | Emergency Room | 21_VA | 512 | 1 | NA |
| 2025-02-23 | Urine | Tradate | Emergency Room | 22_VA* | NA | NA | NA |
| 2025-02-26 | Rectal Swab | Varese | Medicine - 2 | 23_VA* | NA | NA | NA |
| 2025-02-26 | Urine | Varese | Outpatient | 24_VA | 147 | 14 | Major |
| 2025-03-06 | Urine | Varese | Emergency Room | 25_VA | 512 | 1 | NA |
| 2025-03-12 | Rectal Swab | Tradate | Medicine | 26_VA | 512 | - | NA |
| 2025-03-27 | Rectal Swab | Varese | Physical therapy | 27_VA | 147 | 14 | Major |
| 2025-04-01 | Rectal Swab | Varese | Emergency Room | 28_VA | 512 | - | NA |
| 2025-04-28 | Rectal Swab | Varese | Surgery | 29_VA | 147 | 14 | Major |
| 2025-04-29 | RT | Varese | Neurosurgery ICU | 30_VA | 147 | 14 | Major |
| 2025-05-01 | Urine | Tradate | Medicine | 31_VA | 147 | 14 | Major |
| 2025-05-01 | Urine | Tradate | Medicine | 32_VA | 147 | 14 | Major |
| 2025-05-10 | Rectal Swab | Varese | Medicine - 1 | 33_VA | 6668 | 1 | NA |
| 2025-05-16 | RT | Varese | Neurosurgery ICU | 34_VA | 147 | 14 | Major |
| 2025-05-19 | Rectal Swab | Varese | Neurosurgery | 35_VA | 147 | 14 | Major |
| 2025-05-19 | Rectal Swab | Varese | Pneumology | 36_VA* | NA | NA | NA |

**Supplementary Table 1. Main characteristics of the NDM-producing *Klebsiella pneumoniae* isolates from the analyzed period**

Summary of all NDM-producing *K. pneumoniae* isolates identified from the ASST Sette Laghi network between December 2024 and May 2025. Asterisks indicate isolates that could not be resuscitated upon thawing. **ST**: sequence type; **NA**: not assessed.
