# Supplementary material for: Emergence and local dissemination of NDM-14-producing Klebsiella pneumoniae ST147: a phylodynamic and epidemiological investigation
Source: Antimicrob Agents Chemother. 2026 Aug 11;70(9):e00545-26. doi: 10.1128/aac.00545-26 (PMC13712845; doi:10.1128/aac.00545-26)
Supplement: Supplementary file 3 [file aac.00545-26-s0006.docx]

| **ID** | **MEM** | **CZA** | **MVB** | **IMR** | **FDC** | **AZA** | **CIP** | **SXT** | **AMI** | **COL** | **TGC** | **ERV** | **ARG** |
| --- | --- | --- | --- | --- | --- | --- | --- | --- | --- | --- | --- | --- | --- |
| 1_VA | > 8 | > 8 | > 32 | > 8 | 0.5 | 0.06 | > 2 | > 160 | > 32 | <= 0.5 | 0.25 | 0.5 | *aac(6')-Ib-cr, aph(3')-Ia, aph(3')-VI, armA, bla*_CTX-M-15_*, bla*_NDM-14_*, bla*_OXA-1_*, bla*_OXA-9_*, bla*_TEM-1A_*, catB3, dfrA5, mph(A), mph(E), msr(E), qnrS1, sul1, sul2* |
| 3_VA | > 8 | > 8 | > 32 | > 8 | 0.125 | 0.12 | > 2 | > 160 | > 32 | <= 0.5 | 0.25 | 0.25 | *aac(6')-Ib-cr, aph(3')-Ia, aph(3')-VI, armA, bla*_CTX-M-15_*, bla*_NDM-14_*, bla*_OXA-1_*, bla*_OXA-9_*, bla*_TEM-1A_*, catB3, dfrA5, mph(A), mph(E), msr(E), qnrS1, sul1, sul2* |
| 4_VA | > 8 | > 8 | > 32 | > 8 | 1 | 0.03 | > 2 | > 160 | > 32 | <= 0.5 | 0.25 | 0.25 | *aac(6')-Ib-cr, aph(3')-Ia, aph(3')-VI, armA, bla*_CTX-M-15_*, bla*_NDM-14_*, bla*_OXA-1_*, bla*_OXA-9_*, bla*_TEM-1A_*, catB3, dfrA5, mph(A), mph(E), msr(E), qnrS1, sul1, sul2* |
| 6_VA | > 8 | > 8 | > 32 | > 8 | 1 | 0.12 | > 2 | > 160 | > 32 | <= 0.5 | 0.25 | 0.25 | *aac(6')-Ib-cr, aac(6')-Ib, aph(3')-VI, armA, bla*_NDM-14_*, bla*_OXA-1_*, bla*_OXA-9_*, bla*_TEM-1A_*, catB3, mph(E), msr(E), qnrS1, sul1, sul2* |
| 7_VA | > 8 | > 8 | > 32 | > 8 | 2 | 0.12 | > 2 | > 160 | > 32 | <= 0.5 | 0.25 | 0.25 | *aac(6')-Ib, aph(3')-Ia, aph(3')-VI, armA, bla*_CTX-M-15_*, bla*_NDM-14_*, bla*_OXA-1_*, bla*_OXA-9_*, bla*_TEM-1A_*, catB3, dfrA5, mph(A), mph(E), msr(E), qnrS1, sul1, sul2* |
| 8_VA | > 8 | > 8 | > 32 | > 8 | 0.5 | 0.06 | > 2 | > 160 | > 32 | <= 0.5 | 0.5 | 0.5 | *aph(3')-VI, armA, bla*_CTX-M-15_*, bla*_NDM-1_*, dfrA5, mph(A), mph(E), msr(E), qnrS1, sul1, sul2* |
| 10_VA | > 8 | > 8 | > 32 | > 8 | 2 | 0.12 | > 2 | > 160 | > 32 | <= 0.5 | 0.5 | 0.25 | *aac(6')-Ib-cr, aph(3')-Ia, armA, bla*_CTX-M-15_*, bla*_NDM-14_*, bla*_OXA-1_*, bla*_OXA-9_*, bla*_TEM-1A_*, catB3, dfrA5, mph(A), mph(E), msr(E), qnrS1, sul1, sul2* |
| 13_VA | > 8 | > 8 | > 32 | > 8 | 2 | 0.12 | > 2 | > 160 | > 32 | <= 0.5 | 0.25 | 0.25 | *aac(6')-Ib-cr, aph(3')-Ia, aph(3')-VI, armA, bla*_CTX-M-15_*, bla*_NDM-14_*, bla*_OXA-1_*, bla*_OXA-9_*, bla*_TEM-1A_*, catB3, dfrA5, mph(A), mph(E), msr(E), qnrS1, sul1, sul2* |
| 14_VA | > 8 | > 8 | > 32 | > 8 | 1 | 0.06 | > 2 | > 160 | > 32 | <= 0.5 | 0.25 | 0.25 | *aac(6')-Ib, aph(3')-Ia, aph(3')-VI, armA, bla*_CTX-M-15_*, bla*_NDM-14_*, bla*_OXA-1_*, bla*_OXA-9_*, bla*_TEM-1A_*, catB3, dfrA5, mph(A), mph(E), msr(E), qnrS1, sul1, sul2* |
| 17_VA | > 8 | > 8 | > 32 | > 8 | 0.5 | 0.03 | > 2 | > 160 | > 32 | <= 0.5 | 0.25 | 0.5 | *aph(3')-VI, armA, bla*_CTX-M-15_*, bla*_NDM-14_*, bla*_OXA-1_*, bla*_OXA-9_*, bla*_TEM-1A_*, catB3, dfrA5, mph(A), mph(E), msr(E), qnrS1, sul1, sul2* |
| 20_VA | > 8 | > 8 | > 32 | > 8 | 2 | 0.03 | > 2 | > 160 | > 32 | <= 0.5 | 0.25 | 0.5 | *96.86, aph(3')-Ia, aph(3')-VI, armA, bla*_CTX-M-15_*, bla*_NDM-14_*, bla*_OXA-1_*, bla*_OXA-9_*, bla*_TEM-1A_*, catB3, dfrA5, mph(A), mph(E), msr(E), qnrS1, sul1, sul2* |
| 24_VA | > 8 | > 8 | > 32 | > 8 | 4 | 0.12 | > 2 | > 160 | > 32 | <= 0.5 | 0.5 | 0.25 | *aac(6')-Ib, aph(3')-Ia, aph(3')-VI, armA, bla*_CTX-M-15_*, bla*_NDM-14_*, bla*_OXA-1_*, bla*_OXA-9_*, bla*_TEM-1A_*, catB3, dfrA5, mph(A), mph(E), msr(E), qnrS1, sul1, sul2* |
| 27_VA | > 8 | > 8 | > 32 | > 8 | 2 | 0.06 | > 2 | > 160 | > 32 | <= 0.5 | 0.25 | 0.25 | *aac(6')-Ib, aph(3')-Ia, aph(3')-VI, armA, bla*_CTX-M-15_*, bla*_NDM-14_*, bla*_OXA-1_*, bla*_OXA-9_*, bla*_TEM-1A_*, catB3, dfrA5, mph(A), mph(E), msr(E), qnrS1, sul1, sul2* |
| 29_VA | > 8 | > 8 | > 32 | > 8 | 2 | 0.06 | > 2 | > 160 | > 32 | <= 0.5 | 0.25 | 0.25 | *aac(6')-Ib, aph(3')-Ia, aph(3')-VI, armA, bla*_CTX-M-15_*, bla*_NDM-14_*, bla*_OXA-1_*, bla*_OXA-9_*, bla*_TEM-1A_*, catB3, dfrA5, mph(A), mph(E), msr(E), qnrS1, sul1, sul2* |
| 30_VA | > 8 | > 8 | > 32 | > 8 | 1 | 0.06 | > 2 | > 160 | > 32 | <= 0.5 | 0.25 | 0.25 | *aac(6')-Ib-cr, aph(3')-Ia, aph(3')-VI, armA, bla*_CTX-M-15_*, bla*_NDM-14_*, bla*_OXA-1_*, bla*_OXA-9_*, bla*_TEM-1A_*, catB3, dfrA5, mph(A), mph(E), msr(E), qnrS1, sul1, sul2* |
| 31_VA | > 8 | > 8 | > 32 | > 8 | 0.25 | 0.06 | > 2 | > 160 | > 32 | <= 0.5 | 0.25 | 0.5 | *aac(6')-Ib, aph(3')-Ia, aph(3')-VI, armA, bla*_CTX-M-15_*, bla*_NDM-14_*, bla*_OXA-1_*, bla*_OXA-9_*, bla*_TEM-1A_*, catB3, dfrA5, mph(A), mph(E), msr(E), qnrS1, sul1, sul2* |
| 32_VA | > 8 | > 8 | > 32 | > 8 | 0.5 | 0.06 | > 2 | > 160 | > 32 | <= 0.5 | 0.25 | 0.5 | *aac(6')-Ib, aph(3')-Ia, aph(3')-VI, armA, bla*_CTX-M-15_*, bla*_NDM-14_*, bla*_OXA-1_*, bla*_OXA-9_*, bla*_TEM-1A_*, catB3, dfrA5, mph(A), mph(E), msr(E), qnrS1, sul1, sul2* |
| 34_VA | > 8 | > 8 | > 32 | > 8 | 1 | 0.12 | > 2 | > 160 | > 32 | <= 0.5 | 0.5 | 0.25 | *aac(6')-Ib-cr, aph(3')-Ia, aph(3')-VI, armA, bla*_CTX-M-15_*, bla*_NDM-14_*, bla*_OXA-1_*, bla*_OXA-9_*, bla*_TEM-1A_*, catB3, dfrA5, mph(A), mph(E), msr(E), qnrS1, sul1, sul2* |
| 35_VA | > 8 | > 8 | > 32 | > 8 | 1 | 0.12 | > 2 | > 160 | > 32 | <= 0.5 | 0.25 | 0.25 | *aac(6')-Ib-cr, aph(3')-Ia, aph(3')-VI, armA, bla*_CTX-M-15_*, bla*_NDM-14_*, bla*_OXA-1_*, bla*_OXA-9_*, bla*_TEM-1A_*, catB3, dfrA5, mph(A), mph(E), msr(E), qnrS1, sul1, sul2* |

**Supplementary Table 2 Antimicrobial susceptibility profiles of the NDM-producing ST147 *Klebsiella pneumoniae***

Minimum inhibitory concentrations (MICs in µg/L) were determined by broth microdilution and, for AZA, a gradient strip test. **MEM**: Meropenem; **CZA**: Ceftazidime-Avibactam; **MVB**: Meropenem-Vaborbactam; **IMR**: Imipenem-Relebactm; **FDC**: Cefiderocol; **AZA**: Aztreonam-Avibactam; **CIP**: Ciprofloxacin; **SXT**: Trimethoprim-Sulfamethoxazole; **AMI**: Amikacin; **COL**: Colistin; **TGC**: Tigecycline; **ERV**: Eravacycline; **ARG**: Antimicrobial Resistance Gene
